# High levels of unfavourable treatment outcomes in children with drug-sensitive TB in Sierra Leone

**DOI:** 10.5588/pha.25.0046

**Published:** 2026-03-06

**Authors:** N. Sesay, I.F. Kamara, A.M.V. Kumar, P. Thekkur, A.A. Alwani, B.D. Fofanah, A.R.Y. Kamara, A. Bah, L. Farma-Grant, M.A. Sesay, W.K. Lahai, J.A. Koroma, S.M. Tengbe, F. Kanu, G. Ameh, S.M. Kanneh, R. Zachariah, M. Mahmoud

**Affiliations:** 1 Government of Sierra Leone, Ministry of Health, University of Sierra Leone Teaching Hospital Complex, Ola During Children’s Hospital, Freetown, Sierra Leone;; 2 World Health Organization Country Office, Freetown, Sierra Leone;; 3 Centre for Operational Research, International Union Against Tuberculosis and Lung Disease, Paris, France;; 4 Yenepoya Medical College, Yenepoya (Deemed to be University), Mangaluru, India;; 5 Government of Sierra Leone, Ministry of Health, Freetown, Sierra Leone;; 6 Government of Sierra Leone, Ministry of Health, Directorate of National Child Health Programme, Ministry of Health, Freetown, Sierra Leone;; 7 Government of Sierra Leone, Ministry of Health, Directorate of National Leprosy and Tuberculosis Programme, Ministry of Health, Freetown, Sierra Leone;; 8 UNICEF, UNDP, World Bank, WHO Special Programme for Research and Training in Tropical Diseases (TDR), Geneva, Switzerland.

**Keywords:** tuberculosis, operational research, SORT IT, unsuccessful outcomes, mortality, universal health coverage

## Abstract

**SETTING:**

Ola During Children’s Hospital, a tertiary-level paediatric facility affiliated with a university and located in Freetown, Sierra Leone. No published studies from Sierra Leone have evaluated treatment outcomes in children (<15 years) with drug-sensitive TB (DS-TB).

**OBJECTIVE:**

To assess compliance with national TB treatment guidelines and evaluate treatment outcomes among children with DS-TB.

**DESIGN:**

A non-concurrent cohort study, utilising routinely collected secondary patient data from TB treatment master cards. Poisson regression was done to calculate adjusted relative risks (aRR).

**RESULTS:**

Of 689 children, 95% received treatment regimens compliant with national guidelines. However, only 32% achieved favourable outcomes, while 68% had unfavourable outcomes (7% death, 30% loss to follow-up, 31% not evaluated). HIV co-infection (aRR = 1.2) and HIV-unknown status (aRR = 1.5), residence outside urban areas (aRR = 1.3), and extra-pulmonary TB (aRR = 1.2) were significantly associated with unfavourable outcomes. Children treated in 2023 (aRR = 0.7) and 2024 (aRR = 0.6) had better outcomes than those in 2022.

**CONCLUSION:**

Despite high compliance with treatment protocols, paediatric TB outcomes were alarmingly unfavourable. Strengthening follow-up systems and data recording, integrating TB-HIV services, and decentralising care are critical to improving outcomes in this vulnerable population.

TB is a preventable and curable disease; yet, it is the world’s leading cause of death from a single infectious agent.^1^ In children, especially those under the age of 5 years, TB is often underdiagnosed due to atypical presentations of the disease, placing them at higher risk of severe disease and unfavourable treatment outcomes.^2,3^ In low- and middle-income countries, additional drivers of the TB epidemic in children include poverty, malnutrition, HIV co-infection, and a high prevalence of TB among adults.^2,4,5^ Sub-Saharan Africa (SSA) carries a disproportionate TB burden, with an estimated 800,000 children affected annually.^6,7^ In Sierra Leone, an estimated 24,000 people contract TB yearly, and it is the sixth leading cause of death, with an estimated 1,770 annual fatalities – a case fatality ratio of 7.4%. Approximately 1,536 children under 15 years with TB are reported annually, accounting for 7% of all cases notified in 2023.^1^ However, this figure is likely to be an underestimate due to diagnostic challenges and weaknesses in the monitoring and evaluation system in health facilities.^8,9^

The most recent WHO TB report revealed a global treatment success of 88% for new drug-sensitive TB (DS-TB) cases treated with first-line drugs.^1^ However, this figure is lower in SSA with 79% treatment success.^7^ More alarming is that treatment success among children is lowest in Africa at 70%.^7,10^ Sierra Leone’s National Tuberculosis Control Program aims to increase the early detection of paediatric TB and increase the treatment success.^9^ However, there has been no formal analysis of treatment outcomes in children under 15 years of age. Ensuring high TB treatment success in children requires high compliance with recommended treatment regimens and addressing factors linked to unsuccessful outcomes. Achieving this goal necessitates surveillance and operational research.^11–13^ A review of existing literature revealed that studies focused on treatment outcomes in adults and in those with drug-resistant TB.^9,14–16^ However, there is a lack of studies focused on children. To bridge this gap, we assessed i) compliance with national TB treatment guidelines and ii) treatment outcomes in a cohort of children with DS-TB initiated on treatment from January 2022 to June 2024 at one of the largest paediatric referral centres in Sierra Leone.

Insights from this study can inform improvements in clinical practice, strengthen health system capacity, and guide strategies aimed at addressing unfavourable outcomes, such as mortality, treatment failure, and loss to follow-up.^16,17^

## METHODS

This was a non-concurrent cohort study utilising routinely collected, secondary data in TB treatment cards.

### Study setting

Sierra Leone, a low-income country located on the Atlantic coast of West Africa between Guinea and Liberia, has a population of over 8 million people, with children (<15 years) accounting for 41% of the total.^17^ The capital, Freetown, hosts the majority of the country’s tertiary and specialised health care facilities, including paediatric care. The study was conducted at Ola During Children’s Hospital (ODCH), Sierra Leone’s oldest paediatric facility and part of the University of Sierra Leone Teaching Hospital complex. It provides free comprehensive paediatric, HIV, and DS-TB care, serving predominantly the Western Area while referring drug-resistant TB cases to specialised centres.

### TB programme framework at ODCH

TB diagnosis involves a combination of clinical assessment based on medical history and physical examination, chest radiography, and laboratory tests such as sputum smear microscopy for acid-fast bacilli, Xpert MTB/RIF/ultra, and culture. Clinically diagnosed TB includes both cases confirmed through clinical assessment alone and those supported by radiological evaluation. Chest X-ray findings were extracted when available; however, they were not consistently recorded for all patients. Children diagnosed with DS-TB receive a 2-month intensive phase (IP) treatment. Severely ill patients are admitted for directly observed therapy, followed by a caregiver-administered continuation phase (CT) at home under caregiver supervision, with periodic hospital visits for medication collection.

HIV and antiretroviral therapy (ART) status were extracted retrospectively from routine TB treatment records, specifically the patient TB treatment cards, which served as the sole data source. Missing or undocumented ART information was classified as ‘not recorded’ and analysed as not on ART.

Standardised TB treatment regimens for children are tailored by disease site and weight band.^18^ Children under 15 years and weighing under 25 kg with pulmonary or extra-pulmonary TB (EPTB) (excluding TB meningitis, miliary TB, and osteoarticular TB) receive a 2-month IP of rifampicin/isoniazid/pyrazinamide/ethambutol (RHZE – 75/50/150/100 mg), followed by a 4-month CP of rifampicin/isoniazid (RH – 75/50 mg). For children with TB meningitis, miliary TB, or osteoarticular TB, the CP extends to 10 months. The number of fixed-dose combination (FDC) tablets administered daily ranges from one to four, depending on the child’s weight (4–7, 8–11, 12–15, 16–24 kg). Children less than 4 kg receive half a tablet. Children weighing 25 kg and above receive adult dosing: those between 25 and 39 kg receive two tablets and those between 40 and 54 kg receive three tablets of RHZE (150/75/400/275 mg) in the IP and rifampicin/isoniazid (150/75 mg) in the CP, respectively.

### Study population

Children (<15 years) with DS-TB, who were initiated on first-line treatment at ODCH between 1 January 2022 and 30 June 2024, were included.

### Data collection

Data were collected by trained health care professionals experienced in patient chart review. To ensure uniformity, all data collectors underwent standardised training on the data collection tool, the use of Epicollect5, and how to collect the data from treatment cards. The data were collected from 1 March to 30 April 2025. The principal investigator provided overall supervision and conducted routine cross-checks to ensure data accuracy, without disrupting clinical services.

### Statistical analysis

Data from Epicollect5 were exported and analysed using Stata 16.1 (StataCorp LLC, College Station, TX, USA). Demographic and clinical characteristics of the study participants, non-compliance with treatment regimens, and treatment outcomes were summarised using frequencies and proportions. Patients were classified into favourable (cured and treatment completed) and unfavourable outcome (treatment failure, death, loss to follow-up, and not evaluated) groups following national and WHO guidelines.^18,19^ Compliance with the three recommended paediatric regimens for DS-TB was assessed by evaluating whether the prescribed regimen and duration matched with the TB type and whether the dosage (based on tablet count) was appropriate for the child’s weight. To identify factors associated with unfavourable treatment outcomes, both unadjusted and adjusted generalised linear models (Poisson regression) were employed. Adjusted relative risks (aRRs) with 95% confidence intervals (CIs) were calculated to quantify the associations.

### Ethical statement

Ethics approval was granted by the Sierra Leone Ethics and Scientific Review Committee (SLESRC – 026/02/2025). Hospital management authorised data access, and informed consent was waived as the study involved only secondary data review.

## RESULTS

There were 689 children with DS-TB during the study period (Table 1). The majority (548, 79.5%) were under the age of five. More than half (372, 54%) were males. Most children (440, 63.9%) lived in urban areas. Pulmonary TB was predominant (81%), while EPTB accounted for 11.5% of children. Among EPTB cases (*n* = 79), TB meningitis (37, 46.7%) and TB lymphadenitis (34, 43%) were most common. Nearly one third (206, 29.9%) of the children were HIV-positive, and among them, only 22.8% were on ART.

**TABLE 1.** Baseline characteristics and treatment outcomes of children with drug-sensitive TB initiated on treatment at Ola During Children’s Hospital, Sierra Leone, from January 2022 to June 2024 (*n* = 689).

Characteristics	n (%)^A^
Age (in years)	
0–4	548 (79.5)
5–9	95 (13.8)
10–14	46 (6.7)
Gender	
Men	372 (54.0)
Women	317 (46.0)
Year of treatment initiation	
2022	311 (45.1)
2023	277 (40.2)
2024	101 (14.7)
Residence	
Western Area Urban	440 (63.9)
Western Area Rural	221 (32.1)
Other provinces	28 (4.0)
TB type	
Pulmonary TB	559 (81.1)
Extra-pulmonary TB	79 (11.5)
Not recorded	51 (7.4)
Site of EPTB (*N* = 79)	
Lymph node	34 (43.0)
Abdomen	1 (1.3)
Meningitis	37 (46.8)
Osteoarticular	6 (7.6)
Pericardial	1 (1.3)
Type of TB diagnosed	
Bacteriologically confirmed	190 (27.6)
Clinically diagnosed	483 (70.1)
Not recorded	16 (2.3)
Patient type	
New	565 (82.0)
Previously treated	32 (4.6)
Not recorded	92 (13.4)
HIV status	
Positive	206 (29.9)
Negative	447 (64.9)
Unknown	1 (0.1)
Not recorded	35 (5.1)
ART status (*N* = 206)	
On ART	47 (22.8)
Not on ART	2 (1.0)
Not recorded	157 (76.2)
Treatment guideline	
Compliant	652 (94.6)
Non-compliant	37 (5.4)
Treatment outcome	
Treatment completed	213 (30.9)
Cured	9 (1.3)
Death	48 (7.0)
Lost to follow-up	206 (29.9)
Not evaluated	213 (30.9)

ART = antiretroviral therapy; EPTB = extra-pulmonary TB.

A Column percentage.

### Treatment guidelines compliance

Compliance with treatment guidelines was high, with 652 (94.6%) compliant with intensive-phase prescriptions (Figure). Non-compliance was primarily due to prescription of incorrect doses – 5 children had underdosing (one FDC tablet less than that recommended per day) and 32 children had overdosing (one FDC tablet more than that recommended per day).

**FIGURE.**
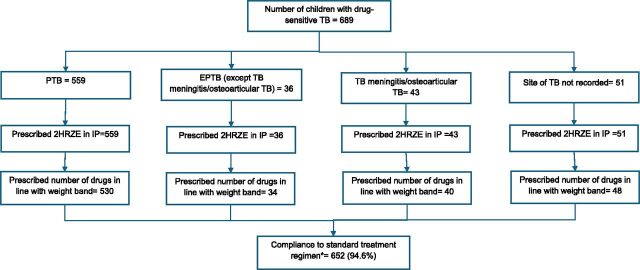
Compliance to treatment guidelines among children with drug-sensitive TB initiated on treatment at Ola During Children’s Hospital, Sierra Leone, from January 2022 to June 2024. *PTB and EPTB patients prescribed 2HRZE in the intensive phase with the number of drugs per day in line with the weight bands. HRZE = isoniazid/rifampicin/pyrazinamide/ethambutol; IP = intensive phase; PTB = pulmonary TB; EPTB = extra-pulmonary TB.

### Treatment outcomes and factors associated with unfavourable outcomes

Of the 689 children, 222 (32.2%) had favourable outcomes (cured 1.3%; treatment completed 30.9%) and 467 (67.8%) had unfavourable outcomes (death 7%; loss to follow-up 29.9%; and not evaluated 30.9%) (Table 1). Treatment success was 46.6% after excluding patients classified as ‘not evaluated’ (Table 2). Mortality was notably high in children with TB meningitis (15/37, 40.5%). Children treated in 2023 (aRR = 0.7, 95% CI: 0.6–0.7) and 2024 (aRR = 0.6, 95% CI: 0.5–0.7) had a significantly lower risk of unfavourable treatment outcomes compared to those treated in 2022 (Table 3). Children residing in provincial areas had a significantly higher likelihood of unfavourable outcomes compared with those from Western Area Urban (aRR = 1.3, 95% CI: 1.1–1.5). HIV-positive children had a higher risk of unfavourable outcomes compared to HIV-negative children (aRR = 1.2, 95% CI: 1.0–1.3). Children with an unknown HIV status had the highest risk, with all cases (100%) classified as having unfavourable outcomes (aRR = 1.5, 95% CI: 1.4–1.7). EPTB was associated with higher risk (aRR = 1.2, 95% CI: 1.0–1.3) compared to pulmonary TB.

**TABLE 2.** Treatment outcomes excluding ‘not evaluated’.

Outcome	Number	Proportion (including ‘not evaluated’)	Proportion (excluding ‘not evaluated’)
Total	689		
Treatment success	222	32.2	46.6
Cured	9	1.3	1.9
Treatment completed	213	30.9	44.7
Unfavourable outcomes	467	67.8	53.4
Death	48	7.0	10.1
Loss to follow-up	206	29.9	43.3
Not evaluated	213	30.9	NA

**TABLE 3.** Demographic and clinical characteristics associated with the unfavourable treatment outcomes among children with drug-sensitive TB initiated on treatment at Ola During Children’s Hospital, Sierra Leone, from January 2022 to June 2024.

Characteristics	Total	Unsuccessful outcomes^A^	RR (95% CI)	aRR (95% CI)^C^
		*n* (%)^B^		
Total, *n*	689	467 (67.8)		
Age (in years)				
0–4	548	379 (69.2)	Ref	Ref
5–9	95	53 (65.4)	0.9 (0.8–1.1)	1.0 (0.8–1.1)
10–14	46	35 (58.3)	0.9 (0.7–1.1)	1.0 (0.8–1.2)
Gender				
Men	372	248 (66.7)	Ref	Ref
Women	317	219 (69.1)	1.0 (0.9–1.1)	1.0 (0.9–1.1)
Year of treatment initiation				
2022	311	262 (82.2)	Ref	Ref
2023	277	157 (56.7)	0.7 (0.6–0.8)	0.7 (0.6–0.7)
2024	101	48 (47.5)	0.6 (0.5–0.7)	0.6 (0.5–0.7)
Residence				
Western Area Urban	440	281 (63.9)	Ref	Ref
Western Area Rural	221	160 (72.4)	1.1 (1.0–1.3)	1.1 (1.0–1.2)
Other	28	26 (92.9)	1.5 (1.3–1.6)	1.3 (1.1–1.5)
Site of TB infection				
Pulmonary TB	559	372 (66.5)	Ref	Ref
Extra-pulmonary TB	79	61 (77.2)	1.2 (1.0–1.3)	1.2 (1.0–1.3)
Not recorded	51	34 (66.7)	1.0 (0.8–1.2)	1.0 (0.9–1.3)
Type of TB diagnosed				
Bacteriologically confirmed	190	131 (68.9)	Ref	Ref
Clinically diagnosed	483	324 (67.1)	1.0 (0.9–1.1)	1.0 (0.9–1.1)
Not recorded	16	12 (75.0)	1.1 (0.8–1.5)	0.9 (0.7–1.2)
Patient type				
New	565	378 (66.9)	Ref	Ref
Previously treated	32	22 (64.8)	1.1 (0.3–4.6)	1.2 (0.3–4.7)
Not recorded	92	67 (72.8)	0.8 (0.3–2.2)	0.7 (5–2.9)
HIV status				
Positive	206	151 (73.3)	1.2 (1.0–1.3)	1.2 (1.0–1.3)
Negative	447	280 (62.6)	Ref	Ref
Unknown	36	36 (100.0)	1.6 (1.5–1.7)	1.5 (1.4–1.7)
Compliance to IP prescription				
Yes	635	434 (68.3)	Ref	Ref
No	54	33 (61.0)	0.9 (0.7–1.1)	0.9 (0.7–1.1)

RR = relative risk; aRR = adjusted relative risk; CI = confidence interval; IP = intensive phase.

A Unfavourable outcomes include lost to follow-up, death, failure, and not evaluated.

B Row percentage.

C Generalised linear model using modified Poisson regression.

## DISCUSSION

This is the first study from Sierra Leone presenting a comprehensive analysis of treatment outcomes among children with DS-TB over a 30-month period. We found a high proportion of unfavourable outcomes (67.8%), which are among the highest reported globally, despite high compliance with national treatment guidelines. Excluding ‘not evaluated’ cases, treatment success was only 46.6%. The outcomes were worse in certain subgroups, such as children with HIV co-infection, those with EPTB, and those residing outside the urban areas. This highlights critical gaps in paediatric TB care and health system performance.

One of the most striking findings is the disconnect between high compliance with treatment guidelines (94.6%) and unfavourable treatment outcomes. This suggests that adherence to standardised regimens alone is insufficient to ensure successful treatment in paediatric populations. While correct dosing and regimen selection are foundational, they must be complemented by robust systems for follow-up, treatment support, and management of comorbidities such as HIV. The high loss to follow-up (29.9%) and not evaluated cases (30.9%) point to systemic weaknesses in continuity of care, particularly during the CT when treatment is administered by the caregiver at home. This also reflects a lack of capacity and resources at ODCH for active follow-up of children and weaknesses in systematic transfer out of children to referral centres and related documentation. In contrast, in a large study from Ethiopia of children with DS-TB, the treatment success rate was 92.2% and death was 2.8%; loss to follow-up was only about 1.1%, and about 0.4% of all children were not evaluated.^20^ This may be due to differences in geographical coverage, patient characteristics, socio-economic conditions, and programme capacities.

HIV co-infection was a significant predictor of unfavourable outcomes, with only 22.8% of HIV-positive children documented as receiving ART despite national guidelines recommendation. Incomplete HIV/ART documentation reflects weak TB-HIV integration, leading to missed opportunities for universal testing, timely ART initiation, and coordinated care. The 100% unfavourable outcomes among children with unknown HIV status underscore the critical importance of universal HIV testing and accurate documentation. These findings align with previous studies that have demonstrated the detrimental impact of HIV on TB treatment outcomes.^21–23^

Extra-pulmonary TB, particularly TB meningitis, was associated with the highest mortality and poorest outcomes, consistent with global evidence that severe paediatric TB forms have substantially lower treatment success than pulmonary TB. A study in Mozambique showed EPTB was associated with worse outcomes, and HIV-positive status further worsened the outcome.^24^ The fact that ODCH is a referral centre seeing more severe TB cases may explain the disproportionate representation of TB meningitis and EPTB, as well as the elevated mortality outcomes.

Children residing outside Western Area Urban had significantly higher risks of unfavourable outcomes. This disparity likely reflects inequities in access to health care, transportation barriers, and socio-economic challenges faced by families in rural and remote areas. While ODCH serves as a national referral centre, the burden of travel and follow-up may be prohibitive for families from distant regions.

Encouragingly, treatment outcomes improved over time, with children treated in 2023 and 2024 showing significantly lower risks of unfavourable outcomes compared to those treated in 2022. These improved treatment outcomes may reflect strengthened programmatic practices, including staff training on documentation by the TB programme, introduction of a more user-friendly treatment register, and enhanced monitoring and evaluation. Although the exact cause is uncertain, these improvements likely supported better data completeness, guideline adherence, and patient management. Another commendable finding is the high compliance with treatment guidelines, with nearly all children being treatment compliant at the start of treatment. A limitation of this assessment, however, is that we could not assess the compliance as the treatment progressed, as changes in weight and doses were not documented in the treatment cards.

Our study has several strengths. It is one of the first to systematically assess paediatric DS-TB treatment outcomes in Sierra Leone, addressing a critical gap in the literature. The large sample size and use of standardised data collection tools enhanced the reliability of findings. The use of adjusted Poisson regression models provided robust estimates of associations between clinical and demographic factors and treatment outcomes. We reported the study findings in line with STROBE (Strengthening the Reporting of Observational Studies in Epidemiology) guidelines.^25^ There were also some limitations. First, we relied on routinely collected secondary data, which may be subject to documentation errors and missing information. The high proportion of ‘not evaluated’ outcomes and unrecorded variables (e.g., ART status) reflects these challenges. Second, the study was conducted at a single tertiary hospital, which may limit generalisability nation-wide. Third, we did not collect data on nutritional status and other comorbidities, which may influence outcomes. Finally, the absence of a qualitative component limited the exploration of underlying reasons for the unfavourable treatment outcomes.

Despite these limitations, the findings of this study highlight several areas for targeted intervention:

i) Strengthening the monitoring of the CT of treatment: Potential strategies include systematically documenting transfer-outs, recording caregiver contact information, conducting follow-up calls after 15 days, and allocating dedicated staff for patient follow-up.
ii) Integration of TB-HIV services: Ensuring that all patients with TB are tested for HIV, and if HIV-positive, children are promptly initiated on ART and monitored closely. Co-location of TB and HIV services, joint training of staff, and shared data systems could facilitate integration.
iii) Decentralisation and outreach: Expanding paediatric TB services to peripheral health facilities and enhancing outreach to primary care units and sharing patient information with peripheral facilities can improve care continuity and reduce geographic disparities in outcomes. Implementing a web-based or online TB register accessible to receiving facilities would facilitate real-time tracking, reduce loss to follow-up, and ensure timely monitoring.
iv) Strengthening data recording: The high proportion of cases classified as ‘not evaluated’ points to weaknesses in data capture and reporting. Training staff in documentation and routine data audits could improve accountability and programme performance.

## CONCLUSION

The study reveals alarmingly unfavourable outcomes among children with DS-TB in Sierra Leone, despite high compliance with national treatment guidelines. HIV co-infection, residence outside urban areas, and EPTB were significant predictors of unfavourable outcomes. These findings call for urgent programmatic action to strengthen follow-up systems, integrate TB-HIV care, decentralise services, and improve data quality. Addressing these gaps is essential to achieving the national goal of improved paediatric TB outcomes and contributing to the global End TB strategy.
